# Patient characteristics and valuation changes impact quality of life and satisfaction in total knee arthroplasty – results from a German prospective cohort study

**DOI:** 10.1186/s12955-019-1237-3

**Published:** 2019-12-09

**Authors:** Julia Felix, Christian Becker, Matthias Vogl, Peter Buschner, Werner Plötz, Reiner Leidl

**Affiliations:** 1 German Research Center for Environmental Health, Institute for Health Economics and Health Care Management, Helmholtz Zentrum München, Postfach 1129, 85758 Neuherberg, Germany; 2 Krankenhaus Barmherzige Brüder München, Akademisches Lehrkrankenhaus der Technischen Universität München, Romanstraße 93, 80639 Munich, Germany; 3 Klinikum rechts der Isar, Technical University Munich, 81675 Munich, Germany; 4 Institute for Health Economics and Health Care Management and Munich Center of Health Sciences, Ludwig-Maximilians-University, Ludwigstr. 28 RG, 80539 Munich, Germany

**Keywords:** Germany, Total knee arthroplasty, Preoperative patient characteristics, Health-related quality of life, Satisfaction, Response shift, External valuation

## Abstract

**Background:**

Evaluation of variations in pre- and postoperative patient reported outcomes (PRO) and the association between preoperative patient characteristics and health and satisfaction outcomes after total knee arthroplasty (TKA) may support shared decision-making in Germany. Since previous research on TKA health outcomes indicated valuation differences in longitudinal data, experienced-based population weights were used for the first time as an external valuation system to measure discrepancies between patient and average population valuation of HRQoL.

**Methods:**

Baseline data (*n* = 203) included sociodemographic and clinical characteristics and PROs, measured by the EQ-5D-3 L and WOMAC. Six-month follow-up data (*n* = 161) included medical changes since hospital discharge, PROs and satisfaction. A multivariate linear regression analysis was performed to evaluate the relationship between preoperative patient characteristics and PRO scores. Patient acceptable symptom state (PASS) was calculated to provide a satisfaction threshold. Patient-reported health-related quality of life (HRQoL) valuations were compared with average experienced-based population values to detect changes in valuation.

**Results:**

One hundred thirty-seven subjects met inclusion criteria. All PRO measures improved significantly. Preoperative WOMAC and EQ-5D VAS, housing situation, marital status, age and asthma were found to be predictors of postoperative outcomes. 73% of study participants valued their preoperative HRQoL higher than the general population valuation, indicating response shift. Preoperatively, patient-reported EQ-5D VAS was substantially higher than average experienced-based population values. Postoperatively, this difference declined sharply.

Approximately 61% of the patients reported satisfactory postoperative health, being mainly satisfied with results if postoperative WOMAC was ≥82.49 (change ≥20.25) and postoperative EQ-5D VAS was ≥75 (change ≥6).

**Conclusion:**

On average, patients benefited from TKA. Preoperative WOMAC and EQ-5D VAS were predictors of postoperative outcomes after TKA. Particularly patients with high absolute preoperative PRO scores were more likely to remain unsatisfied. Therefore, outcome prediction can contribute to shared-decision making. Using general population valuations as a reference, this study underlined a discrepancy between population and patient valuation of HRQoL before, but not after surgery, thus indicating a potential temporary response shift before surgery.

## Background

Total knee arthroplasty (TKA) is a widely used surgical procedure [1, 2]. In 2014, Germany’s incidence of TKA was 197 per 100,000 inhabitants, which was the second highest rate in Europe [2]. While operation rates in Germany have stabilized at a high level, the rate of knee replacements worldwide continues to increase [2, 3]. Considering the increasing number of knee replacements and corresponding high costs [2, 3], knowledge about potential TKA outcomes is becoming increasingly important.

Although TKA is generally considered cost-effective [3, 4] and improved function, mobility and quality of life [1, 3, 5, 6], a subset of patients report prolonged pain, functional impairment [7] or remain dissatisfied with outcomes [8–12].

To better understand patients’ needs and reasons for poor outcomes regarding health and satisfaction, patient reported outcome (PRO) measures, such as health-related quality of life (HRQoL) or disease-specific measures, are highly relevant [13]. PROs support patients and clinicians in shared decision-making, which may improve outcomes [13] and could reduce costs [9]. Although there is evidence that preoperative patient characteristics [14–16] have an impact on HRQoL and satisfaction with surgery after TKA [17, 18], evidence in Germany is lacking.

Aside from measurable patient characteristics, psychological phenomena could influence HRQoL before and after TKA. Previous research comparing pre- and postsurgical TKA health outcomes showed changes in HRQoL valuation over time, which was termed response shift [19, 20]. Response shift describes the change of an individual’s internal standards of measurement (scale recalibration), values (reprioritization) or definition (reconceptualization) regarding a theoretical construct like HRQoL [21, 22]. Health issues, like illness, treatment or other life events, require adaptation of behavioral, cognitive and affective processes and subsequently cause a change in health state [21, 23]. Response shift may influence pre- and postoperative valuation of health states and thus, HRQoL change due to surgical treatment which may affect conclusions about treatment effects [23]. Therefore, response shift must be considered when analyzing surgical treatment outcomes.

The first aim of this paper is to identify the factors influencing patient-reported health and satisfaction outcomes after TKA among German patients. Thus, the association between preoperative patient characteristics and TKA outcomes is evaluated and a satisfaction threshold is calculated. Secondly, the paper assessed pre- and postoperative PRO variation and compared patient-reported HRQoL valuations with average population weights. These are used as an external valuation reference to identify eventual discrepancies between the patient’s valuation and this reference at different time points.

## Material and methods

### Study design and population

The study was conducted as a single-center cohort study recruiting TKA patients over 6 months at a teaching hospital with 365 beds in Munich, Germany. A total of 203 patients who had a TKA between January 2012 and June 2012 completed the preoperative health examination survey. Baseline data, including sociodemographic and clinical characteristics and PRO measures were assessed before TKA at hospital admission. Follow-up data, comprising medical changes since hospital discharge, PROs and satisfaction with TKA, were collected 6 months after hospital discharge between July 2012 and December 2012. All individuals who agreed with study participation were included. Patients were excluded from the analysis if they had a revision of TKA, a further operation of the operated knee or incomplete PRO data. The study was approved by the ethics commission of Klinikum rechts der Isar, Technical University Munich (ethical vote no. 5140/11). Informed consent was obtained from all participants included in the study.

### Sociodemographic and clinical characteristics

Sociodemographic characteristics included age at operation, sex, marital status, housing situation and insurance status. Baseline clinical variables included height, weight, primary diagnosis, comorbidities, American Society of Anesthesiologists (ASA) Classification, Charlson Comorbidity Index, operations and procedures, type of arthroplasty (cemented or hybrid), number of operations on affected joint before TKA, preoperative and postoperative hemoglobin, number of transfused erythrocyte concentrates used, discharge type, and Knee Society Score. Follow-up data on newly diagnosed thrombosis, embolism, myocardial infarction, infection, further operations on affected joint or other relevant medical events were collected 6 months after discharge.

### PROs

#### EQ-5D-3 L

Generic pre-and postoperative HRQoL was determined using the EQ-5D-3 L, which includes a descriptive part of five dimensions with three problem levels and a visual analogue scale (VAS), ranging from 0 (worst health state) to 100 (best health state), in which study participants evaluate their current health state [24]. Descriptive EQ-5D outcomes were weighted with the German experienced health state (EHS) EQ-5D value set. The German EHS-based value set is based on the general population’s valuation of their own health state and thus, should reflect patient-reported EQ-5D VAS [25]. This value set has been proven valid in chronic diseases, including hip arthroplasty [26, 27]. It was also shown to have better psychometric properties than a utility-based value set, to predict patients VAS and to also detect the impact of health shocks such as myocardial infarction on valuation response [28, 29]. The EHS-based index weights describe the average health state valuation in the general population. Given the above properties, it is used here as an external valuation reference. The population average valuations were compared with the patient-reported EQ-5D VAS, thus enabling detection of valuation changes.

#### WOMAC

To investigate patient reported knee-specific outcomes, the Western Ontario and McMaster Universities Arthritis (WOMAC) Index, a reliable and valid [30, 31] HRQoL measure to investigate knee-specific PROs, was used. The WOMAC index is calculated based on 24 questions regarding pain, stiffness and mobility [31]. We used a Likert scale version of the WOMAC score with answers ranging from 0 to 10. To facilitate comparison between different WOMAC dimensions and other outcomes, the scores were transformed to a 0 (worst) to 100 (best) scale, as recently recommended [32].

### Satisfaction

Patient satisfaction was assessed 6 months after TKA using a 0 (not satisfied) to 10 (very satisfied) Likert scale. We defined a cut-off value of ≥9 as being satisfied, which is consistent with previous research [33].

### Data analysis

Descriptive statistics of socioeconomic variables, clinical characteristics and preoperative, postoperative and change WOMAC and EQ-5D VAS scores (postoperative score minus preoperative score) were calculated. We addressed possible non-response bias by comparing responders in the follow-up measurement to non-responders, and included study participants to excluded participants, using Chi-squared and Fisher exact test (for variables with *n* < 5) for categorical data and Mann-Whitney U-test for continuous data. Correlation between EQ-5D VAS, EHS-based index and WOMAC index was analyzed using Pearson correlation coefficients ρ. Spearman rank correlation was computed to assess the association between postoperative and changes in EQ-5D VAS and WOMAC scores and satisfaction-score.

Patient acceptable symptom state (PASS) was calculated to identify absolute postoperative and changes in EQ-5D VAS and WOMAC cut-offs related to patient satisfaction with TKA. PASS is defined as the threshold beyond which patients consider themselves well [17, 34, 35]. To identify PASS, we used two different statistical approaches based on previous literature [33, 35]. First, a receiver operating characteristic (ROC) curve was plotted for postoperative WOMAC and EQ-5D VAS. PASS was estimated as EQ-5D VAS or WOMAC score that performed best with regard to Youden Index [36]. Secondly, we used the approach by Tubach et al. [35] to validate ROC curve results, which involved constructing a cumulative distribution function of satisfied patients and selecting the lowest EQ-5D VAS or WOMAC value that was achieved by 75% of satisfied study participants.

The strength of the relationship between preoperative patient characteristics and postoperative WOMAC and EQ-5D VAS scores was evaluated by conducting a multivariate linear regression analysis. Predictive variables were selected using stepwise selection (SLE = 0.3, SLS = 0.10), significance level was defined as α = 0.05. To rule out possible multicollinearity among predictive variables, we examined variance inflation factors (VIF) [37, 38]. To reveal discrepancies between the patient’s valuation and the average population reference, each before and after surgery, mean EQ-5D VAS scores were compared with mean EHS-based index values. Study subjects were then divided into four groups according to preoperative and postoperative difference between VAS and EHS-based value set and compared with regard to satisfaction with surgery, using a Chi-squared test. All data analyses were conducted with Microsoft Excel 2016 (Microsoft Corporation, Redmond; WA, USA) and SAS 9.4 (SAS Institute Inc., Cary, NC, USA).

## Results

In total, 161 (79.31%) of 203 study participants completed the 6-months follow-up examination. Health characteristics and preoperative EQ-5D and WOMAC values did not significantly differ between patients who completed follow-up and those who were lost to follow-up. However, patients lost to follow-up were significantly younger (*p = 0.04*), and were less often discharged to inpatient rehabilitation (*p = 0.02*) (Additional file 1: Table S1). Of those who completed follow-up, 137 patients met our inclusion criteria and were analysed. Excluded study participants were more frequently females (*p* = 0.03), persons with compulsory insurance (*p* = 0.03), previous TKA (*p* = 0.02), and lower knee function score (*p* = 0.03) and were less often discharged to inpatient rehabilitation (*p = 0.03*; see Additional file 2: Table S2).

### Health outcomes

Demographics and preoperative health characteristics of all study participants are presented in Table 1. Additional details about clinical characteristics are provided in Additional file 3: Table S3.

**Table 1:** Descriptive statistics and preoperative clinical characteristics of the study population

	N/Mean (SD)	%
n	137	
Age	70.15 (8.76)	
Gender male	53	38.69
BMI (Mean)	28.95 (5.78)	
BMI ≥ 30	55	39.86
Metabolic syndrome (yes)	7	5.11
Marital status		
Married	82	59.85
Single	13	9.49
Divorced	8	5.84
Living Apart	1	0.73
Widowed	33	24.09
Housing situation		
Alone	45	34.35
With partner	54	41.22
With family	31	23.66
Other	1	0.76
Health insurance		
Compulsory	69	50.36
Private	68	49.64
Major diagnosis		
Right	75	54.74
Left	61	44.53
Bilateral	1	0.73
Operations at joint before TKR		
0	82	59.85
1	39	28.47
2	12	8.76
> 3	4	2.92
Cement (cement or hybrid)		
Cement	66	48.18
Already TKR	14	10.22
Already THR	14	10.22
Discharge		
Home	26	18.98
Inpatient rehabilitation	112	81.02
Charlson Comorbidity Index		
0	89	64.96
1	36	26.28
2	5	3.65
> 3	7	5.11
ASA Physical Score Classification		
1	33	24.09
2	86	62.77
3	18	13.14
Infiltration anaesthesia	44	32.12
FNB/ASNB/SSNB	64	46.72
PDA	8	5.84
Preoperative hemoglobin	13.94 (1.14)	
Number of operations and other procedures	2.00 (0.97)	
Knee Society Score	52.33 (16.23)	
Knee Society Score function	66.24 (20.99)	

*Abbreviations*: *FNB* femoral nerve block, *ASNB* anterior sciatic nerve block, *SSNB* subgluteal sciatic nerve block, *PDA* peridural anaesthesia

Average preoperative, postoperative and change in EQ-5D and WOMAC scores improved during the observation period (Table 2). The preoperative difference between EHS-based EQ-5D Index and EQ-5D VAS diminished postoperatively. Changes in individual EQ-5D dimensions are shown in Additional file 4: Table S4. Subjectively, approximately 4% of all participants described their health state as worse, 13% as similar, 30.5% as better and 48% as much better after TKA than before surgery (Additional file 5: Table S5).

**Table 2:** Descriptive statistics of patient reported outcomes (PROs)

PROs	pre-operative		6 months post OP		change	
	Mean	SE	Mean	SE	Mean	SE
WOMAC pain	55.04	1.54	85.75	1.18	30.72	1.66
WOMAC stiffness	46.64	2.00	75.26	1.61	28.61	2.34
WOMAC function	53.94	1.69	82.70	1.26	28.75	1.75
WOMAC sum	53.56	1.61	81.23	1.25	27.67	1.74
EHS-based value set	52.37	1.31	71.69	1.40	19.32	1.61
EQ-5D VAS	62.17	1.66	73.00	1.47	10.83	2.07

Preoperative, postoperative and change EQ-5D VAS, EHS-based value set and WOMAC index were significantly correlated, except for postoperative VAS and preoperative WOMAC index and postoperative VAS and preoperative VAS.

After stepwise selection using all preoperative patient characteristics, the final multivariate regression model included age, marital status divorced, housing situation with family, WOMAC function score, Knee Society Score, EQ-5D VAS, Asthma and other arthroplasty (Table 3).

**Table 3:** Multivariate linear regression models predicting change in WOMAC Sum and change in EQ-5D VAS

Preoperative values	Change in WOMAC Sum (adj. R^2^ = 0.65)		Change in EQ-5D VAS (adj. R^2^ = 0.58)	
	Estimate [95% CI]	VIF	Estimate [95% CI]	VIF
Intercept	66.66^***^ [59.09–78.95]		40.59^**^ [14.43–66.74]	
Age			0.46^*^ [0.09–0.83]	1.08
Marital status – divorced	−13.11^*^ [−23.06 - -3.17]	1.04	−13.13^*^ [−25.82 - -0.43]	1.03
Housing situation – with family	7.47^*^ [1.36–13.59]	1.05		
WOMAC function score	−0.78^***^ [−0.93 - -0.62]	1.66		
Knee Society Score function	−0.22^**^ [−0.37 - -0.08]	1.57		
EQ-5D VAS	0.26^***^ [0.11–0.40]	1.38	−0.97^***^ [−1.13 - -0.81]	1.05
J 45 - Asthma			−16.34^**^ [−27.71 - -4.98]	1.04
5–829 – other arthroplasty	10.60 [− 0.98–22.18]	1.02		

^*^p < 0.05 ^**^p < 0.01 ^***^p < 0.001

About 73% of patients rated their preoperative EQ-5D VAS higher than the EHS-based value set. Postoperatively this number decreased to about 62%. There was no significant association between pre- and postoperative difference between EQ-5D VAS and EHS-based value set and satisfaction (*X*^*2*^ *= 4.60, df = 3, p = 0.2022*) (Additional file 6: Table S6). A more detailed description of patient distribution regarding the difference between EQ-5D VAS and value set is shown in the appendix (Additional file 6: Table S6).

### Satisfaction outcomes and PASS

Comparing changes in WOMAC and EQ-5D VAS to preoperative scores showed that patients with lower preoperative scores had higher absolute changes and reached change satisfaction thresholds more often (Additional file 9: Figure S1). Postoperatively, satisfied study participants had a higher mean change in WOMAC score of 32.7 than unsatisfied patients (mean change of 20.9). The mean change in EQ-5D VAS of satisfied patients was 14.6, but only 6.8 for unsatisfied patients. In total, almost 61% of all patients stated their postoperative health as satisfying.

Using Spearman’s rank correlation, we found a significant correlation between satisfaction and change in EQ-5D VAS (*r*_*s*_ *= .177, p = .039, n = 137)*, change in WOMAC index (*r*_*s*_ *= .298, p = .0004, n = 137)*, postoperative EQ-5D VAS (*r*_*s*_ *= .502, p < .0001, n = 137)* and postoperative WOMAC scores (*r*_*s*_ *= .489, p = .0001, n = 137)*.

**Fig. 1:**
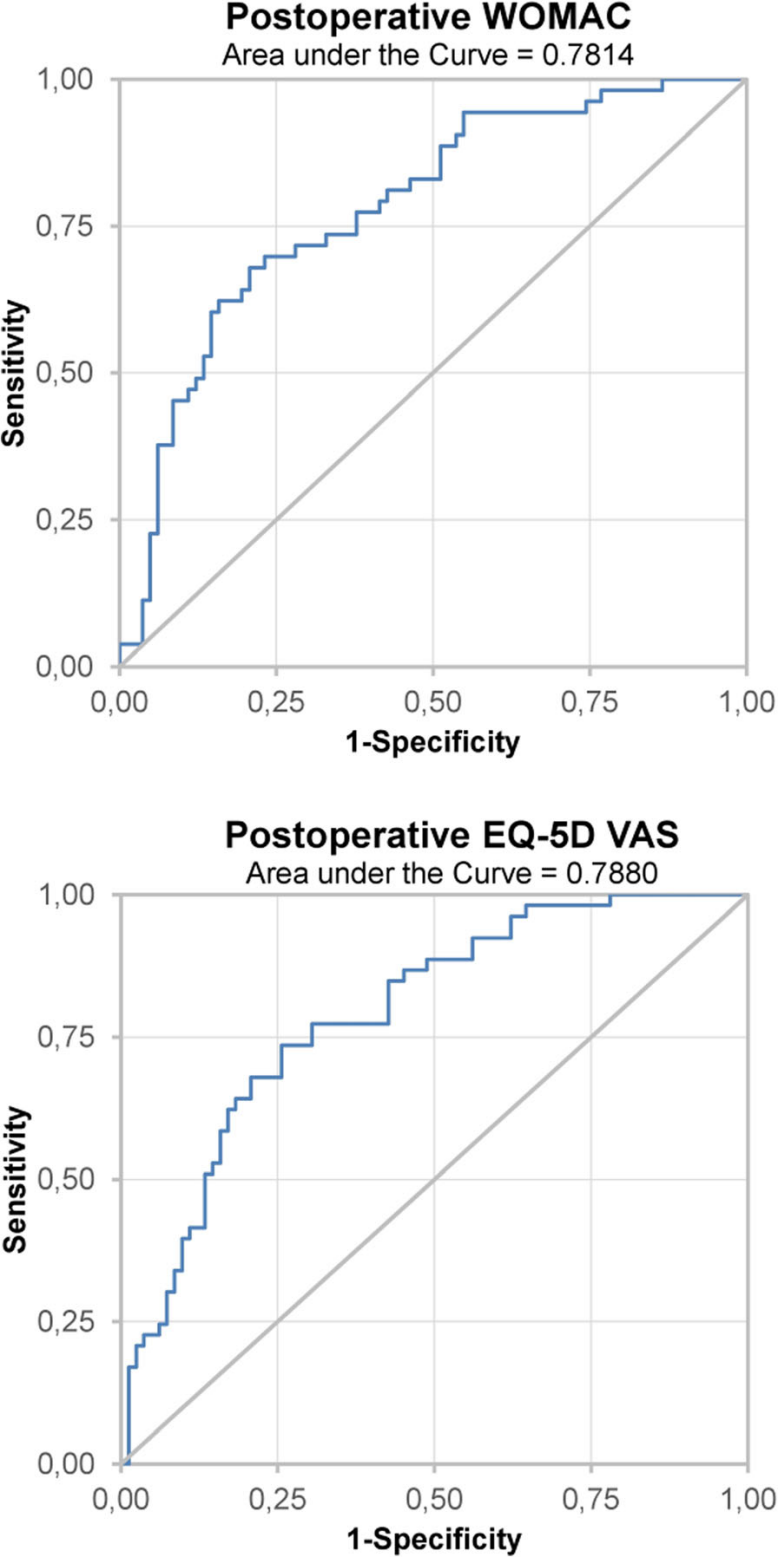
ROC curve using satisfaction and postoperative WOMAC and EQ-5D VAS scores

Both methods for calculating PASS yielded similar results. PASS as calculated based on ROC curves was 82.49 for postoperative WOMAC, 20.25 for change in WOMAC, 75 for postoperative EQ-5D VAS and 6 for change in EQ-5D VAS (Fig. 1).

**Fig. 2:**
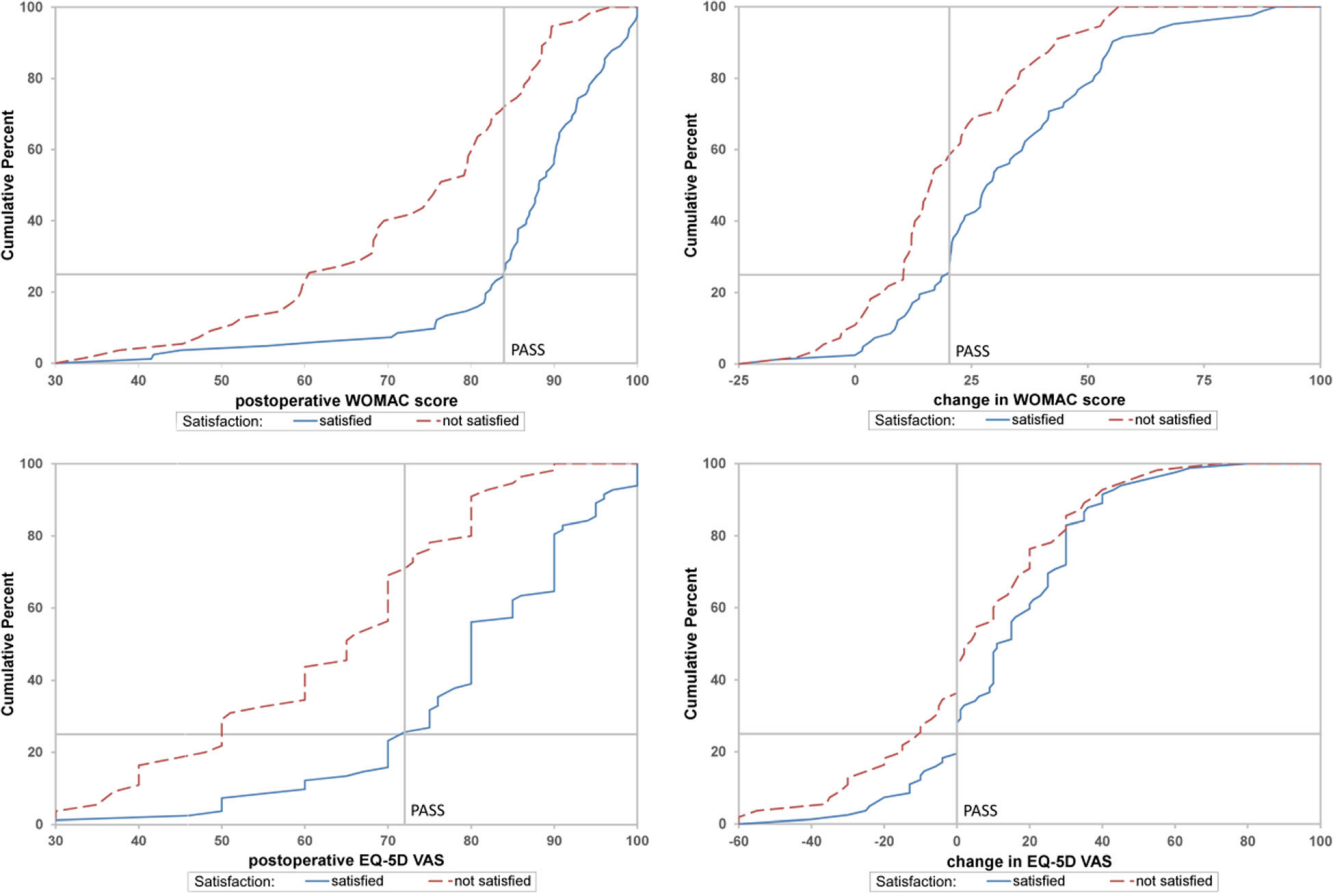
Cumulative distribution function to investigate PASS using the approach by Tubach et al. [35]. Among satisfied patients 75% assessed their postoperative WOMAC score above 83.96 and their change in WOMAC score higher than 20.25. Postoperative EQ-5D VAS was rated higher than 72.00 and EQ-5D VAS change above 0.00 by 75% of patients who considered their health state as satisfactory

PASS estimates, considering only satisfied patients, were 83.96 [95% CI 80.75–85.61] for postoperative WOMAC, 20.25 [95% CI 11.91–21.12] for WOMAC change, 72.00 [95% CI 70.00–76.00] for postoperative EQ-5D VAS and at least 0.00 [95% CI -6.00 – 6.00] for EQ-5D change (Fig. 2).

There was no significant difference in socio-demographic patient characteristics between satisfied and unsatisfied patients (Additional file 7: Table S7). However, patients who reached PASS change thresholds had average preoperative EQ-5D and WOMAC scores of 57.06 and 46.04 respectively compared to 77.65 and 65.12 for patients who did not reach PASS thresholds. The average preoperative EQ-5D and WOMAC scores of patients who reached postoperative PASS thresholds were 64.87 and 56.74, while patients who remained unsatisfied had average scores of 58.48 and 47.46.

## Discussion

The evaluation of pre- and postoperative PRO variation and the association between preoperative patient characteristics and health and satisfaction outcomes in TKA patients in Germany revealed several interesting results. All PRO measures improved significantly. Preoperative WOMAC and EQ-5D VAS, housing situation, marital status, age and asthma were found to be predictors of postoperative outcomes. 73% of study participants valued their preoperative HRQoL higher than the general population valuation. Preoperatively, patient-reported EQ-5D VAS was substantially higher than average experienced-based population values. Postoperatively, this difference declined sharply. Approximately 61% stated their postoperative health as satisfying, being mainly satisfied with results if postoperative WOMAC was ≥82.49 (change ≥20.25) and postoperative EQ-5D VAS was ≥75 (change ≥6).

### Preoperative patient characteristics and health outcomes

Mean WOMAC and EQ-5D improved 6 months after TKA, supporting previous research findings [14, 17, 39, 40]. However, 4% of all participants described their health state as worse and 13% as similar.

Multivariate analysis of determinants of PRO change indicated that marital status “divorced” had a negative impact on WOMAC change while living with family had a positive impact on change of WOMAC and EQ-5D VAS. This is consistent with previous research showing social support to positively affect outcomes in patients with joint replacement surgery [41, 42]. Furthermore, our results corroborated findings of other studies that age does not negatively affect TKA outcomes [6, 14]. However, the effect of age on TKA outcomes is controversially discussed [43, 44]. Our findings that higher preoperative PRO scores predicted lower change in PROs also confirm earlier research results of total joint arthroplasty patients [33, 39].

### Comparison of patient-reported EQ-5D VAS and EHS-based population valuation

The difference between patient-reported EQ-5D VAS and EHS-based valuation, particularly for preoperative values was interesting. On average, patients in our study sample overrated their preoperative health state compared to the general German population. This difference diminished substantially postoperatively. Previous research in TKA patients found different HRQoL value decrements for the same health problems pre- and postoperatively indicating response shift. Postoperative value decrements were larger in all reported studies [19, 45, 46]. As the German EHS-based value set has been shown to be a good prediction for HRQoL valuation [28, 29] we assume that the valuation differences indicate eventual response shift. Using external valuations as a reference could therefore be a new methodological approach to identify possible response shift.

Pickard et al. [45] hypothesized that larger HRQoL value decrements are a possible result of dissatisfaction with surgery results. Our study results did not support this conclusion as we did not find a significant relation between satisfaction and differences between pre- and postoperative EQ-5D VAS and EHS-based value sets according to a Chi-squared test (Additional file 6: Table S6). Razmjou et al. [20] and Zhang et al. [19] demonstrated that TKA patients preoperatively judged themselves better than they did when asked again postoperatively. However, these study results were based on the then-test method, which is susceptible to recall bias [47]. Our study results also indicate response shift preoperatively, as the difference between the average patient-reported EQ-5D VAS and EHS-based index weights decreased postoperatively, supporting previous research results. Response shift in terms of preoperative valuation discrepancy between patient’s valuation and average population valuation leads to smaller effects of surgery. An important question for future research is whether, and if so, when, valuation changes have taken place prior to TKA and hospital admission.

An additional regression analysis on the impact of the difference between patient-reported EQ-5D VAS and EHS-based valuation on postoperative WOMAC sum indicated that high patient-reported values compared to average population values correlate positively with knee specific outcomes (Additional file 8: Table S8). If relatively high, pre-operative VAS reports by patients would be interpreted as a sign of optimism, this result would resemble improved outcomes found for optimistic patients after hip replacement [48].

### Satisfaction outcomes and PASS

Approximately 61% of the study population were subjectively satisfied with TKA surgery results. Satisfaction rates found in previous research on TKA surgery were often higher, yet these are not directly comparable because of different examination methods or definition of satisfaction [17, 49]. PASS thresholds of this study confirmed findings of previous research [50], although other studies analyzed other PRO measures [49] or subscales instead of overall scores [51, 52]. Regarding PASS thresholds, we found a difference between preoperative EQ-5D (57.06) and WOMAC (46.04) scores in patients who reached the threshold compared to those not reaching the threshold (preoperative EQ-5D: 77.65, WOMAC: 65.12), which indicates that patients with particularly high preoperative PRO scores are more likely to remain unsatisfied after TKA. This insight and the link of PASS thresholds to preoperative PRO scores in general may support clinicians and patients in decision-making and could therefore potentially contribute to shared-decision making and patient-centered care. Satisfaction was significantly associated with change of EQ-5D VAS, change of WOMAC index, postoperative EQ-5D VAS and postoperative WOMAC scores. Although the associations are weak, our results on the role of satisfaction correspond to previous research [53, 54].

### Strengths and limitations

To the best of our knowledge, this is the first study to use a combination of the WOMAC as a knee-specific PRO measure and the generic EQ-5D-3 L to analyse the impact of preoperative patient characteristics on postoperative TKA outcomes and associated PASS thresholds in Germany. Furthermore, the present study adds to the literature by comparing preoperative and postoperative patient-reported EQ-5D VAS and EHS-based population valuation weights to detect discrepancies between patient-valued and average population-valued HRQoL, potentially providing a new methodological approach to detect response shift. A further strength of this study is the breadth of preoperative patient characteristics taken into account.

We are aware that our study has several limitations, including the small sample size and the involvement of just one hospital center for patient acquisition. Accordingly, the results may not be representative for the general German population. For generalization of the results, multicenter research with a larger sample size is needed. Another limitation of this study may be the short follow-up of 6 months. Although previous research found the greatest improvements after TKA in the first 3 months [14], pain, function, mobility, and HRQoL continue to improve up to 12 months postoperatively [14, 16, 17]. There is inconsistent evidence if there is a clinically important improvement in the six to 12 months follow-up period after TKA [55]. To allow for long-term outcome prediction a second follow-up period after 12 months or later should be included in upcoming research projects.

Furthermore, the significant differences in age, health insurance and type of discharge between the study population and lost-to follow up patients may have influenced the reported results. While we considered a large range of socio-economic variables, additional variables would have been useful to detect a potential impact of education and income on health and quality of life outcomes. In addition, we could not account for the impact of different postoperative rehabilitation programs on postoperative outcomes. Importantly, as there are no evidence-based rehabilitation guidelines for TKA patients in Germany [56], follow-up treatments might have an impact on postoperative outcomes. Owing to the lack of a comparison with well-established response shift methodologies such as Oorts’s structural equation modeling [57–59] our results only provide an indication of response shift while an explanation by response shift would require further research. Furthermore, our study results do not contain any information about the different components of response shift.

Another limitation could be the cut-off defining satisfied and not satisfied patients as different cut-offs could affect the proportion of satisfied patients. Previous research only found that cut-off definition had only a small impact on PASS calculations of satisfied patients [33, 49]. Furthermore, we did not calculate PASS subgroup values for different preoperative health states due to the small sample size. Since results of both calculating methods were similar, we expect our WOMAC and EQ-5D VAS PASS estimates to be robust. Future research should include PASS calculation for subgroups.

## Conclusion

On average, patients benefited from TKA and improved in pain, stiffness, function and overall HRQoL. Preoperative WOMAC and EQ-5D VAS were predictors of postoperative outcomes after TKA and social support seems to contribute to health outcomes. PASS calculations suggested that patients with particularly high preoperative PRO scores were more likely to remain unsatisfied after TKA. Outcome prediction, based on identifying the impact of preoperative patient characteristics on health and satisfaction outcomes, can contribute to shared-decision making and may help to create realistic expectations, consequently affecting HRQoL and satisfaction outcomes after TKA. Future research projects with longer follow-up periods are needed to better account for respective outcome prediction.

Using general population valuations as a reference, a discrepancy to patient valuations was only seen before but not after surgery, thus indicating response shift prior to surgery. The change in discrepancy between patient and reference valuation of HRQoL affects the size of treatment effect and thus potentially conclusions building upon this. Further research is needed for more detailed explanation. To evaluate the validity of the methodology of comparing patient-reported HRQoL values and EHS-based population weights to detect and quantify response shift, future research should directly compare the results of this approach with established response shift methodologies. PRO data should be collected earlier to take account of the influence of preoperative events which could be response shift catalysts before TKA and hospital admission.

## Supplementary information

**Additional file 1: Table S1.** Comparison of patients with completed follow-up (‘responders’) and lost to follow-up (‘non-responders’).

**Additional file 2: Table S2.** Comparison of included and excluded study participants.

**Additional file 3: Table S3.** Detailed descriptive statistics and preoperative clinical characteristics (*n* > 5) of study population.

**Additional file 4: Table S4.** Changes in EQ-5D dimensions (n).

**Additional file 5: Table S5.** Time until effect on health state, pain and mobility and general effect after 6 months.

**Additional file 6: Table S6.** Detailed description of patient distribution regarding the difference between EQ-5D VAS and value set and satisfaction.

**Additional file 7: Table S7.** Comparison of satisfied and not satisfied patients.

**Additional file 8: Table S8.** Linear regression model predicting postoperative WOMAC Sum.

**Additional file 9: Figure S1.** Scatterplots of WOMAC sum/EQ-5D VAS change/postoperative scores by preoperative scores, grouped by patient satisfaction.

## Data Availability

The dataset analysed during the current study is not publicly available because patient consent in this study did not include provision of data for public file sharing.

## Abbreviations

ASA: American Society of Anesthesiologists
EHS: Experienced health state
EQ-5D: EuroQol five dimension
HRQoL: Health-related quality of life
PASS: Patient acceptable symptom state
ROC: Receiver operating characteristic
TKA: Total knee arthroplasty
VAS: Visual analogue scale
VIF: Variance inflation factors
WOMAC: Western Ontario and McMaster Universities Arthritis

## References

[1] da Silva RR, AAM S, de Sampaio Carvalho Júnior J, Matos MA (2014). Quality of life after total knee arthroplasty: systematic review. Rev Bras Ortop.

[2] OECD (2016). Hip and knee replacement. Health at a Glance: Europe 2016: State of health in the EU cycle.

[3] Krummenauer F, Wolf C, Gunther KP, Kirschner S (2009). Clinical benefit and cost effectiveness of total knee arthroplasty in the older patient. Eur J Med Res.

[4] Kamaruzaman H, Kinghorn P, Oppong R. Cost-effectiveness of surgical interventions for the management of osteoarthritis: a systematic review of the literature. BMC Musculoskelet Disord. 2017;18(1):183. 10.1186/s12891-017-1540-2. 10.1186/s12891-017-1540-2 PMC5424321 28486957

[5] Becker R, Doring C, Denecke A, Brosz M (2011). Expectation, satisfaction and clinical outcome of patients after total knee arthroplasty. Knee Surg Sports Traumatol Arthrosc.

[6] Kane RL, Saleh KJ, Wilt TJ, Bershadsky B (2005). The functional outcomes of total knee arthroplasty. J Bone Joint Surg Am.

[7] Wylde V, Dieppe P, Hewlett S, Learmonth ID (2007). Total knee replacement: is it really an effective procedure for all?. Knee.

[8] Naili JE, Wretenberg P, Lindgren V, Iversen MD, Hedström M, Broström EW. Improved knee biomechanics among patients reporting a good outcome in knee-related quality of life one year after total knee arthroplasty. BMC Musculoskelet Disord. 2017;18(1):122. 10.1186/s12891-017-1479-3. 10.1186/s12891-017-1479-3 PMC5361836 28327133

[9] Dyck BA, Zywiel MG, Mahomed A, Gandhi R, Perruccio AV, Mahomed NN (2014). Associations between patient expectations of joint arthroplasty surgery and pre- and post-operative clinical status. Expert Review of Medical Devices.

[10] Scott CE, Howie CR, MacDonald D, Biant LC (2010). Predicting dissatisfaction following total knee replacement: a prospective study of 1217 patients. J Bone Joint Surg Br.

[11] Noble PC, Conditt MA, Cook KF, Mathis KB (2006). The John Insall award: patient expectations affect satisfaction with total knee arthroplasty. Clin Orthop Relat Res.

[12] Bourne RB, Chesworth BM, Davis AM, Mahomed NN, Charron KD (2010). Patient satisfaction after total knee arthroplasty: who is satisfied and who is not?. Clin Orthop Relat Res.

[13] Black N (2013). Patient reported outcome measures could help transform healthcare. Bmj.

[14] Papakostidou I, Dailiana ZH, Papapolychroniou T, Liaropoulos L, Zintzaras E, Karachalios TS, Malizos KN (2012). Factors affecting the quality of life after total knee arthroplasties: a prospective study. BMC Musculoskelet Disord.

[15] Jiang Y, Sanchez-Santos MT, Judge AD, Murray DW, Arden NK (2017). Predictors of Patient-Reported Pain and Functional Outcomes Over 10 Years After Primary Total Knee Arthroplasty: A Prospective Cohort Study. J Arthroplasty.

[16] Kornilov Nikolai, Lindberg Maren Falch, Gay Caryl, Saraev Alexander, Kuliaba Taras, Rosseland Leiv Arne, Lerdal Anners (2017). Higher physical activity and lower pain levels before surgery predict non-improvement of knee pain 1 year after TKA. Knee Surgery, Sports Traumatology, Arthroscopy.

[17] Naal FD, Impellizzeri FM, Lenze U, Wellauer V, von Eisenhart-Rothe R, Leunig M (2015). Clinical improvement and satisfaction after total joint replacement: a prospective 12-month evaluation on the patients’ perspective. Qual Life Res.

[18] Hirschmann MT, Testa E, Amsler F, Friederich NF (2013). The unhappy total knee arthroplasty (TKA) patient: higher WOMAC and lower KSS in depressed patients prior and after TKA. Knee Surg Sports Traumatol Arthrosc.

[19] Zhang XH, Li SC, Xie F, Lo NN, Yang KY, Yeo SJ, Fong KY, Thumboo J (2012). An exploratory study of response shift in health-related quality of life and utility assessment among patients with osteoarthritis undergoing total knee replacement surgery in a tertiary hospital in Singapore. Value Health.

[20] Razmjou H, Yee A, Ford M, Finkelstein JA (2006). Response shift in outcome assessment in patients undergoing total knee arthroplasty. J Bone Joint Surg Am.

[21] Sprangers MA, Schwartz CE (1999). Integrating response shift into health-related quality of life research: a theoretical model. Soc Sci Med.

[22] Schwartz CE, Ahmed S, Sawatzky R, Sajobi T, Mayo N, Finkelstein J, Lix L, Verdam MG, Oort FJ, Sprangers MA (2013). Guidelines for secondary analysis in search of response shift. Qual Life Res.

[23] Wilson IB (1999). Clinical understanding and clinical implications of response shift. Soc Sci Med.

[24] EuroQol (1990). EuroQol-a new facility for the measurement of health-related quality of life. Health Policy.

[25] Leidl R, Reitmeir P (2011). A value set for the EQ-5D based on experienced health states: development and testing for the German population. Pharmacoeconomics.

[26] Vogl M, Leidl R, Plotz W, Gutacker N (2015). Comparison of pre- and post-operative health-related quality of life and length of stay after primary total hip replacement in matched English and German patient cohorts. Qual Life Res.

[27] Little MHR, Reitmeir P, Peters A, Leidl R (2014). The impact of differences between patient and general population EQ-5D-3L values on the mean tariff scores of different patient groups. Value Health.

[28] Leidl R, Schweikert B, Hahmann H, Steinacker JM, Reitmeir P (2016). Assessing quality of life in a clinical study on heart rehabilitation patients: how well do value sets based on given or experienced health states reflect patients’ valuations?. Health Qual Life Outcomes.

[29] Leidl R, Reitmeir P, König H-H, Stark R (2012). The performance of a value set for the EQ-5D based on experienced health states in patients with inflammatory bowel disease. Value Health.

[30] Roos EM, Klassbo M, Lohmander LS (1999). WOMAC osteoarthritis index. Reliability, validity, and responsiveness in patients with arthroscopically assessed osteoarthritis. Western Ontario and MacMaster universities. Scand J Rheumatol.

[31] McConnell S, Kolopack P, Davis AM (2001). The Western Ontario and McMaster universities osteoarthritis index (WOMAC): a review of its utility and measurement properties. Arthritis Rheum.

[32] Singh J, Sloan JA, Johanson NA (2010). Challenges with health-related quality of life assessment in arthroplasty patients: problems and solutions. J Am Acad Orthop Surg.

[33] Vogl M, Wilkesmann R, Lausmann C, Hunger M, Plotz W (2014). The impact of preoperative patient characteristics on health states after total hip replacement and related satisfaction thresholds: a cohort study. Health Qual Life Outcomes.

[34] Keurentjes JC, Van Tol FR, Fiocco M, So-Osman C, Onstenk R, Koopman-Van Gemert AWMM, Pöll RG, Nelissen RGHH (2014). Patient acceptable symptom states after total hip or knee replacement at mid-term follow-up: thresholds of the Oxford hip and knee scores. Bone Joint Res.

[35] Tubach F, Ravaud P, Baron G, Falissard B, Logeart I, Bellamy N, Bombardier C, Felson D, Hochberg M, van der Heijde D, Dougados M (2005). Evaluation of clinically relevant states in patient reported outcomes in knee and hip osteoarthritis: the patient acceptable symptom state. Ann Rheum Dis.

[36] Youden WJ (1950). Index for rating diagnostic tests. Cancer.

[37] Robinson C, Schumacker RE (2009). Interaction effects: centering, variance inflation factor, and interpretation issues. Mult Linear Regression Viewpoints.

[38] Alin A (2010). Multicollinearity. Wiley Interdiscip Rev: Comput Stat.

[39] Hawker GA, Badley EM, Borkhoff CM, Croxford R, Davis AM, Dunn S, Gignac MA, Jaglal SB, Kreder HJ, Sale JE (2013). Which patients are most likely to benefit from total joint arthroplasty?. Arthritis Rheum.

[40] Jones CA, Pohar S (2012). Health-related quality of life after total joint arthroplasty: a scoping review. Clin Geriatr Med.

[41] Ethgen O, Vanparijs P, Delhalle S, Rosant S, Bruyere O, Reginster JY (2004). Social support and health-related quality of life in hip and knee osteoarthritis. Qual Life Res.

[42] Theiss MM, Ellison MW, Tea CG, Warner JF, Silver RM, Murphy VJ (2011). The connection between strong social support and joint replacement outcomes. Orthopedics.

[43] Townsend LA, Roubion RC, Bourgeois DM, Leonardi C, Fox RS, Dasa V, Pollock GR (2018). Impact of age on patient-reported outcome measures in total knee arthroplasty. J Knee Surg.

[44] Elmallah RD, Jauregui JJ, Cherian JJ, Pierce TP, Harwin SF, Mont MA (2016). Effect of age on postoperative outcomes following total knee arthroplasty. J Knee Surg.

[45] Pickard AS, Hung YT, Lin FJ, Lee TA (2017). Patient experience-based value sets: are they stable?. Med Care.

[46] Razmjou H, Schwartz CE, Yee A, Finkelstein JA (2009). Traditional assessment of health outcome following total knee arthroplasty was confounded by response shift phenomenon. J Clin Epidemiol.

[47] Schwartz CE, Bode R, Repucci N, Becker J, Sprangers MA, Fayers PM (2006). The clinical significance of adaptation to changing health: a meta-analysis of response shift. Qual Life Res.

[48] Balck F, Lippmann M, Jeszenszky C, Gunther KP, Kirschner S (2016). The influence of optimism on functionality after total hip replacement surgery. J Health Psychol.

[49] Judge A, Arden NK, Kiran A, Price A, Javaid MK, Beard D, Murray D, Field RE (2012). Interpretation of patient-reported outcomes for hip and knee replacement surgery: identification of thresholds associated with satisfaction with surgery. J Bone Joint Surg Br.

[50] Kvamme MK, Kristiansen IS, Lie E, Kvien TK (2010). Identification of cutpoints for acceptable health status and important improvement in patient-reported outcomes, in rheumatoid arthritis, psoriatic arthritis, and ankylosing spondylitis. J Rheumatol.

[51] Escobar A., García Pérez L., Herrera-Espiñeira C., Aizpuru F., Sarasqueta C., Gonzalez Sáenz de Tejada M., Quintana J.M., Bilbao A. (2017). Total knee replacement: Are there any baseline factors that have influence in patient reported outcomes?. Journal of Evaluation in Clinical Practice.

[52] Escobar A, Gonzalez M, Quintana JM, Vrotsou K, Bilbao A, Herrera-Espineira C, Garcia-Perez L, Aizpuru F, Sarasqueta C (2012). Patient acceptable symptom state and OMERACT-OARSI set of responder criteria in joint replacement. Identification of cut-off values. Osteoarthr Cartil.

[53] Jacobs CA, Christensen CP (2014). Factors influencing patient satisfaction two to five years after primary total knee arthroplasty. J Arthroplast.

[54] Kwon SK, Kang YG, Kim SJ, Chang CB, Seong SC, Kim TK (2010). Correlations between commonly used clinical outcome scales and patient satisfaction after total knee arthroplasty. J Arthroplast.

[55] Browne JP, Bastaki H, Dawson J (2013). What is the optimal time point to assess patient-reported recovery after hip and knee replacement? A systematic review and analysis of routinely reported outcome data from the English patient-reported outcome measures programme. Health Qual Life Outcomes.

[56] Gülich M, Mittag O, Müller E, Uhlmann A, Brüggemann S, Jäckel WH (2010). Ergebnisse einer analyse der therapeutischen leistungsdaten (KTL-Daten) von 5 838 rehabilitandinnen und Rehabilitanden nach Hüft- bzw. Knieendoprothesenimplantation. Rehabilitation.

[57] Ahmed S, Bourbeau J, Maltais F, Mansour A (2009). The Oort structural equation modeling approach detected a response shift after a COPD self-management program not detected by the Schmitt technique. J Clin Epidemiol.

[58] Oort FJ, Visser MR, Sprangers MA (2005). An application of structural equation modeling to detect response shifts and true change in quality of life data from cancer patients undergoing invasive surgery. Qual Life Res.

[59] Verdam MG, Oort FJ, Sprangers MA (2016). Using structural equation modeling to detect response shifts and true change in discrete variables: an application to the items of the SF-36. Qual Life Res.

