# Supplementary material for: Patient characteristics and valuation changes impact quality of life and satisfaction in total knee arthroplasty – results from a German prospective cohort study
Source: Health Qual Life Outcomes. 2019 Dec 9;17:180. doi: 10.1186/s12955-019-1237-3 (PMC6902559; doi:10.1186/s12955-019-1237-3)
Supplement: Supplementary file 1 — Additional file 1: Table S1. Comparison of patients with completed follow-up (‘responders’) and lost to follow-up (‘non-responders’). [file 12955_2019_1237_MOESM1_ESM.docx]

**Supplementary Table 1** Comparison of patients with completed follow-up (‘responders’) and lost to follow-up (‘non-responders’)

|  |  | **Responders**  **(n=161 (79.21%))** | **Non-Responders (n=42 (20.79%))** | **p-value** |
| --- | --- | --- | --- | --- |
| **Age** |  | 70.52 (8.69) | 66.50 (10.61) | **0.04** |
| **Gender male** |  | 56 (34.78%) | 17 (40.48%) | 0.36 |
| **BMI (Mean)** |  | 28.87 (5.65) | 30.20 (6.41) | 0.20 |
| **BMI ≥30** |  | 62 (38.51%) | 16 (38.10%) | 1.00 |
| **Metabolic syndrome (yes)** |  | 10 (6.21%) | 3 (7.14%) | 0.73 |
| **Marital status** | Married | 93 (57.76%) | 22 (52.38%) | 0.43 |
|  | Single | 13 (8.07%) | 2 (4.76%) |  |
|  | Divorced | 10 (6.21%) | 5 (11.90%) |  |
|  | Living Apart | 1 (0.62%) | 1 (2.38%) |  |
|  | Widowed | 42 (26.09%) | 10 (23.81%) |  |
| **Housing situation** | Alone | 54 (33.54%) | 15 (35.71%) | 0.98 |
|  | With partner | 62 (38.51%) | 16 (38.10%) |  |
|  | With family | 37 (22.98%) | 11 (26.19%) |  |
|  | Other | 1 (0.62%) | 0 |  |
| **Health insurance** | compulsory | 84 (52.17%) | 23 (54.76%) | **0.02** |
|  | private | 75 (46.58%) | 15 (35.71%) |  |
|  | . | 2 (1.24%) | 4 (9.52%) |  |
| **Major diagnosis** | right | 87 (54.04%) | 17 (40.48%) | **0.003** |
|  | left | 71 (44.10%) | 19 (45.24%) |  |
|  | bilateral | 1 (0.62%) | 0 |  |
|  | . | 2 (1.24%) | 6 (14.29%) |  |
| **Operations at joint before TKR** | 0 | 95 (59.01%) | 21 (50.00%) | 0.51 |
|  | 1 | 47 (29.19%) | 18 (42.86%) |  |
|  | 2 | 13 (8.07%) | 2 (4.76%) |  |
|  | ≥3 | 6 (3.72%) | 1 (2.38%) |  |
| **Cement (cement or hybrid)** | cement | 78 (48.45%) | 16 (38.10%) | 0.05 |
| **Already TKR** |  | 21 (13.04%) | 2 (4.76%) | 0.17 |
| **Already THR** |  | 17 (10.56%) | 7 (16.67%) | 0.29 |
| **Discharge** | home | 30 (18.63%) | 9 (21.43%) | **0.02** |
|  | inpatient rehabilitation | 129 (80.12%) | 29 (69.05%) |  |
|  | . | 2 (1.24%) | 4 (9.24%) |  |
| **Charlson Comorbidity Index** | 0 | 103 (63.98%) | 26 (61.90%) | 0.77 |
|  | 1 | 42 (26.09%) | 9 (21.43%) |  |
|  | 2 | 6 (3.73%) | 3 (7.14%) |  |
|  | ≥3 | 8 (4.97%) | 2 (4.76%) |  |
| **ASA Physical Score Classification** | 1 | 37 (22.98%) | 9 (21.43%) | 0.79 |
|  | 2 | 101 (62.73%) | 28 (66.67%) |  |
|  | 3 | 22 (13.66%) | 4 (9.52%) |  |
| **Infiltration anaesthesia** |  | 50 (31.06%) | 14 (33.33%) | 0.85 |
| **FNB/ASNB/SSNB** |  | 75 (46.58%) | 19 (45.24%) | 1.00 |
| **PDA** |  | 12 (7.45%) | 1 (2.38%) | 0.34 |
| **Preoperative hemoglobin** |  | 13.87 (1.11) | 14.02 (1.11) | 0.34 |
| **Number of operations and other procedures** |  | 2.01 (0.93) | 2.11 (0.93) | 0.18 |
| **Knee Society Score** |  | 52.77 (15.96) | 50.05 (13.53) | 0.30 |
| **Knee Society Score function** |  | 64.91 (21.00) | 67.26 (17.85) | 0.67 |
| **EQ-5D value set (preoperative)** |  | 0.52 (0.15) | 0.51 (0.18) | 0.38 |
| **EQ-5D VAS (preoperative)** |  | 62.16 (19.75) | 53.22 (17.98) | 0.95 |
| **WOMAC pain (preoperative)** |  | 55.70 (18.74) | 53.22 (17.98) | 0.19 |
| **WOMAC stiffness (preoperative)** |  | 47.36 (24.73) | 50.00 (26.34) | 0.59 |
| **WOMAC function (preoperative)** |  | 54.08 (20.19) | 50.19 (20.79) | 0.14 |
| **WOMAC sum (preoperative)** |  | 53.75 (19.40) | 50.80 (19.58) | 0.23 |

Note: Non-responders are patients who participated in the pre-surgical measurement but not in the follow-up one. Significant differences regarding health insurance were ignored due to the large share of missing values for non-responders regarding this variable, and so were the significant differences of which side has been treated as no link to quality of life has been obvious.
