# Supplementary material for: Patient characteristics and valuation changes impact quality of life and satisfaction in total knee arthroplasty – results from a German prospective cohort study
Source: Health Qual Life Outcomes. 2019 Dec 9;17:180. doi: 10.1186/s12955-019-1237-3 (PMC6902559; doi:10.1186/s12955-019-1237-3)
Supplement: Supplementary file 2 — Additional file 2: Table S2. Comparison of included and excluded study participants. [file 12955_2019_1237_MOESM2_ESM.docx]

**Supplementary Table 2** Comparison of included and excluded study participants

|  |  | **Study participants included**  **(n=137 (85.09%))** | **Study participants excluded (n=24 (20.79))** | **p-value** |
| --- | --- | --- | --- | --- |
| **Age** (n=159, 137, 22) |  | 70. 15 (8.76) | 72.82 (8.09) | 0.13 |
| **Gender male** |  | 53 (38.69) | 3 (12.50) | **0.03** |
| **BMI (Mean)** (n=158, 137, 21) |  | 28.95 (5.78) | 28.31 (4.78) | 0.79 |
| **BMI ≥30** |  | 54 (39.42%) | 8 (33.33%) | 1.00 |
| **Metabolic syndrome (yes)** |  | 7 (5.11%) | 3 (12.50%) | 0.17 |
| **Marital status** | Married | 82 (59.85%) | 11 (45.83%) | 0.26 |
|  | Single | 13 (9.49%) | 0 (0.00%) |  |
|  | Divorced | 8 (5.84%) | 2 (8.33%) |  |
|  | Living Apart | 1 (0.73%) | 0 (0.00%) |  |
|  | Widowed | 33 (24.09%) | 9 (37.50%) |  |
| **Housing situation** | Alone | 45 (32.85%) | 9 (37.50%) | 0.87 |
|  | With partner | 54 (39.42%) | 8 (8.33%) |  |
|  | With family | 31 (22.63%) | 6 (25.00%) |  |
|  | Other | 1 (0.73%) | 0 (0.00%) |  |
| **Health insurance** | compulsory | 69 (50.36%) | 15 (62.50%) | **0.01** |
|  | private | 68 (49.64%) | 7 (29.17%) |  |
|  | . | 0 (0.00%) | 2 (8.33%) |  |
| **Major diagnosis** | right | 75 (54.74%) | 12 (50.00%) | 0.05 |
|  | left | 61 (44.53%) | 10 (41.67%) |  |
|  | bilateral | 1 (0.73%) | 0 (0.00%) |  |
|  | . | 0 (0.00%) | 1 (8.33%) |  |
| **Operations at joint before TKR** | 0 | 82 (59.85%) | 13 (54.17%) | 0.34 |
|  | 1 | 39 (28.47%) | 8 (33.33%) |  |
|  | 2 | 12 (8.76%) | 1 (4.17%) |  |
|  | ≥3 | 4 (2.92%) | 2 (8.34%) |  |
| **Cement (cement or hybrid)** | cement | 66 (48.18%) | 12 (60.00%) | 0.35 |
| **Already TKR** |  | 14 (10.22%) | 7 (29.17%) | **0.02** |
| **Already THR** |  | 14 (10.22%) | 3 (12.50%) | 0.72 |
| **Discharge** | home | 26 (18.98%) | 4 (16.67%) | **0.03** |
|  | inpatient rehabilitation | 111 (81.02%) | 18 (75.00%) |  |
|  | . | 0 (0.00%) | 2 (8.33%) |  |
| **Charlson Comorbidity Index** | 0 | 89 (64.96%) | 14 (58.33%) | 1.00 |
|  | 1 | 36 (26.28%) | 6 (25.00%) |  |
|  | 2 | 5 (3.65%) | 1 (4.17%) |  |
|  | ≥3 | 7 (5.11%) | 1 (4.17%) |  |
| **ASA Physical Score Classification** | 1 | 33 (24.09%) | 4 (16.67%) | 0.72 |
|  | 2 | 86 (62.77%) | 15 (62.50%) |  |
|  | 3 | 18 (13.14%) | 4 (16.67%) |  |
| **Infiltration anaesthesia** |  | 44 (32.12%) | 6 (25.00%) | 0.63 |
| **FNB/ASNB/SSNB** |  | 64 (46.72%) | 11 (45.83%) | 1.00 |
| **PDA** |  | 8 (5.84%) | 4 (16.67%) | 0.08 |
| **Preoperative haemoglobin** (n=161, 137,24) |  | 13.94 (1.14) | 13.48 (0.78) | 0.05 |
| **Number of operations and other procedures** (n=159, 137, 22) |  | 2.01 (0.97) | 2.05 (0.65) | 0.47 |
| **Knee Society Score** (n=155, 132, 23) |  | 52.33 (16.23) | 55.30 (14.40) | 0.30 |
| **Knee Society Score function** (n=161, 137, 24) |  | 66.24 (20.99) | 57.29 (19.78) | **0.02** |
| **EQ-5D value set (preoperative)** (n=158, 137, 21) |  | 0.52 (0.15) | 0.51 (0.17) | 0.64 |
| **EQ-5D VAS (preoperative)** (n=161, 137, 24) |  | 62.17 (19.48) | 62.08 (21.57) | 0.96 |
| **WOMAC pain (preoperative) )** (n=161, 137, 24) |  | 55.04 (17.97) | 59.50 (22.74) | 0.21 |
| **WOMAC stiffness (preoperative)** (n=159, 137, 22) |  | 46.64 (23.43) | 51.82 (31.94) | 0.41 |
| **WOMAC function (preoperative) )** (n=160, 137, 23) |  | 53.94 (19.76) | 54.91 (23.03) | 0.65 |
| **WOMAC sum (preoperative)** (n=158, 137, 21) |  | 53.56 (18.80) | 55.00 (23.42) | 0.57 |
