# Supplementary material for: Patient characteristics and valuation changes impact quality of life and satisfaction in total knee arthroplasty – results from a German prospective cohort study
Source: Health Qual Life Outcomes. 2019 Dec 9;17:180. doi: 10.1186/s12955-019-1237-3 (PMC6902559; doi:10.1186/s12955-019-1237-3)
Supplement: Supplementary file 3 — Additional file 3: Table S3. Detailed descriptive statistics and preoperative clinical characteristics (n > 5) of study population. [file 12955_2019_1237_MOESM3_ESM.docx]

Supplementary Table 3 Detailed descriptive statistics and preoperative clinical characteristics (n>5) of study population

|  |  |  | **N/Mean (SD)** | **%** |
| --- | --- | --- | --- | --- |
| n |  |  | 137 |  |
| Age |  |  | 70.15 (8.76) |  |
| Gender male |  |  | 53 | 38.69 |
| BMI (Mean) |  |  | 28.95 (5.78) |  |
| BMI ≥30 |  |  | 54 | 39.42 |
| Metabolic syndrome (yes) |  |  | 7 | 5.11 |
| Marital status |  | Married | 82 | 59.85 |
|  |  | Single | 13 | 9.49 |
|  |  | Divorced | 8 | 5.84 |
|  |  | Living Apart | 1 | 0.73 |
|  |  | Widowed | 33 | 24.09 |
| Housing situation |  | Alone | 45 | 32.85 |
|  |  | With partner | 54 | 39.42 |
|  |  | With family | 31 | 22.63 |
|  |  | Other | 1 | 0.73 |
| Operations at joint before TKR |  | 0 | 82 | 59.85 |
|  |  | 1 | 39 | 28.47 |
|  |  | 2 | 12 | 8.76 |
|  |  | ≥3 | 4 | 2.92 |
| Health insurance |  | compulsory | 69 | 50.36 |
|  |  | private | 68 | 49.64 |
| Major diagnosis |  | right | 75 | 54.74 |
|  |  | left | 61 | 44.53 |
|  |  | bilateral | 1 | 0.73 |
| Cement (cement or hybrid) |  | cement | 66 | 48.18 |
| Already TKR |  |  | 14 | 10.22 |
| Already THR |  |  | 14 | 10.22 |
| Discharge |  | home | 26 | 18.98 |
|  |  | inpatient rehabilitation | 112 | 81.02 |
| Charlson Comorbidity Index |  | 0 | 89 | 64.96 |
|  |  | 1 | 36 | 26.28 |
|  |  | 2 | 5 | 3.65 |
|  |  | ≥3 | 7 | 5.11 |
| ASA Physical Score Classification |  | 1 | 33 | 24.09 |
|  |  | 2 | 86 | 62.77 |
|  |  | 3 | 18 | 13.14 |
| Infiltration anaesthesia |  |  | 44 | 32.12 |
| FNB/ASNB/SSNB |  |  | 64 | 46.72 |
| PDA |  |  | 8 | 5.84 |
| Preoperative hemoglobin |  |  | 13.94 (1.14) |  |
| **comorbidities (>5 times in study population)** |  |  |  |  |
| COPD |  |  | 10 | 7.30 |
| Heart failure |  |  | 5 | 3.65 |
| Myocardial infarction + STENT |  |  | 11 | 8.03 |
| Deep venous thrombosis (DVT) |  |  | 14 | 10.22 |
| Restless legs |  |  | 9 | 6.57 |
| Marcumar |  |  | 7 | 5.11 |
| Reflux |  |  | 17 | 12.41 |
| Number of side diagnosis |  |  | 4.08(3.23) |  |
| D62 – acute anemia |  |  | 12 | 8.76 |
| E03 - hypothyroidism |  |  | 21 | 15.33 |
| E11 - diabetes |  |  | 14 | 10.22 |
| E 66 - obesity |  |  | 9 | 6.57 |
| E 78 – lipidemia |  |  | 15 | 10.95 |
| E 79 – purine/pyrimidine metabolism |  |  | 8 | 5.84 |
| E 87 – dysfunction of water/electrolyte balance |  |  | 8 | 5.84 |
| I10 – arterial hypertonicity |  |  | 92 | 67.15 |
| I 25 – ischemic heart disease |  |  | 7 | 5.11 |
| I 48 – atrial fibrillation |  |  | 11 | 8.03 |
| J 45 - asthma |  |  | 10 | 7.30 |
| N18 – chronic kidney disease and related diseases |  |  | 18 | 13.14 |
| T81 – complications of procedures |  |  | 8 | 5.84 |
| Z86 – personal history of certain other diseases |  |  | 8 | 5.84 |
| Z88 – personal history of drug allergy |  |  | 8 | 5.84 |
| Z 91 – personal history of risk-factors, not elsewhere classified |  |  | 12 | 8.76 |
| Z 92 – personal history of medical treatment |  |  | 20 | 14.60 |
| Z 95 – presence of cardiac/vascular implants |  |  | 13 | 9.49 |
| Z 96 – presence of other functional implants |  |  | 23 | 16.79 |
| Operations and other procedures (>5) |  | 1-697 diagnostic arthroscopy | 9 | 6.57 |
|  |  | [5-822 implantation](http://www.icd-code.de/suche/ops/code/5-822.html?sp=S5-822) of an artificial knee joint | 137 | 100 |
|  |  | 5-829 [other](http://www.icd-code.de/suche/ops/code/5-829.html?sp=S5-829) arthroplasty | 6 | 4.38 |
|  |  | 8-800 [whole blood, erythrocyte concentrate and thrombocyte concentrate](http://www.icd-code.de/suche/ops/code/8-800.html?sp=S8-800) transfusion | 7 | 5.11 |
|  |  | [8-919 acute](http://www.icd-code.de/suche/ops/code/8-919.html?sp=S8-919) pain relief | 87 | 63.50 |
|  |  | [8-930 monitoring](http://www.icd-code.de/suche/ops/code/8-930.html?sp=S8-930) | 8 | 5.84 |
| Number of operations and other procedures |  |  | 2.00 (0.97) |  |
| Knee Society Score |  |  | 52.33 (16.23) |  |
| Knee Society Score function |  |  | 66.24 (20.99) |  |

Abrreviations: FNB – femoral nerve block; ASNB – anterior sciatic nerve block; SSNB – subgluteal sciatic nerve block; PDA – peridural anaesthesia
