# Supplementary material for: Patient characteristics and valuation changes impact quality of life and satisfaction in total knee arthroplasty – results from a German prospective cohort study
Source: Health Qual Life Outcomes. 2019 Dec 9;17:180. doi: 10.1186/s12955-019-1237-3 (PMC6902559; doi:10.1186/s12955-019-1237-3)
Supplement: Supplementary file 4 — Additional file 4: Table S4. Changes in EQ-5D dimensions (n). [file 12955_2019_1237_MOESM4_ESM.docx]

Supplementary Table 4 Changes in EQ-5D dimensions (n)

|  | Before TKR | **6 months follow-up** | | | |
| --- | --- | --- | --- | --- | --- |
|  |  | no | some | severe | total |
| **EQ-5D mobility** | no | 28 | 6 | 0 | 34 |
|  | some | 66 | 37 | 0 | 103 |
|  | severe | 0 | 0 | 0 | 0 |
|  | total | 94 | 43 | 0 | 137 |
| **EQ-5D self-care** | no | 108 | 4 | 1 | 113 |
|  | some | 17 | 3 | 1 | 21 |
|  | severe | 1 | 2 | 0 | 3 |
|  | total | 126 | 9 | 2 | 137 |
| **EQ-5D usual activity** | no | 33 | 7 | 0 | 40 |
|  | some | 59 | 31 | 2 | 92 |
|  | severe | 1 | 4 | 0 | 5 |
|  | total | 93 | 42 | 2 | 137 |
| **EQ-5D pain/discomfort** | no | 2 | 4 | 0 | 6 |
|  | some | 34 | 44 | 2 | 80 |
|  | severe | 13 | 36 | 2 | 51 |
|  | total | 49 | 84 | 4 | 137 |
| **EQ-5D anxiety/depression** | no | 93 | 9 | 0 | 102 |
|  | some | 20 | 12 | 2 | 34 |
|  | severe | 0 | 1 | 0 | 1 |
|  | total | 113 | 22 | 2 | 137 |
