# Supplementary material for: Patient characteristics and valuation changes impact quality of life and satisfaction in total knee arthroplasty – results from a German prospective cohort study
Source: Health Qual Life Outcomes. 2019 Dec 9;17:180. doi: 10.1186/s12955-019-1237-3 (PMC6902559; doi:10.1186/s12955-019-1237-3)
Supplement: Supplementary file 5 — Additional file 5: Table S5. Time until effect on health state, pain and mobility and general effect after 6 months. [file 12955_2019_1237_MOESM5_ESM.docx]

Supplementary Table 5 Time until effect on health state, pain and mobility and general effect after 6 months

| **Weeks until effect** |  | **Health state (SD)** | **Pain (SD)** | **Mobility (SD)** |
| --- | --- | --- | --- | --- |
| weeks mean (SD) |  | 9.54(6.12) | 6.71(5.76) | 8.38(5.61) |
| n (Missings) |  | 115 (22) | 116 (21) | 108 (29) |
| not yet better |  | 11 (8.73) | 9 (7.20) | 16 (12.90) |
| n (Missings) |  | 126 (11) | 125 (12) | 124 (13) |
| **Effect 6 month post OP** |  | **n (%)** | **n (%)** | **n(%)** |
| Much worse |  | 0 | 0 | 0 |
| Worse |  | 6 (4.69) | 3 (2.19) | 6 (4.38) |
| Similar |  | 15 (11.72) | 12 (8.76) | 25 (18.25) |
| Better |  | 48 (37.50) | 35 (25.55) | 39 (28.47) |
| Much Better |  | 59 (46.09) | 77 (56.20) | 58 (42.34) |
| no information |  | 9 (6.57) | 10 (7.30) | 1. (6.57) |
