# Supplementary material for: Patient characteristics and valuation changes impact quality of life and satisfaction in total knee arthroplasty – results from a German prospective cohort study
Source: Health Qual Life Outcomes. 2019 Dec 9;17:180. doi: 10.1186/s12955-019-1237-3 (PMC6902559; doi:10.1186/s12955-019-1237-3)
Supplement: Supplementary file 6 — Additional file 6: Table S6. Detailed description of patient distribution regarding the difference between EQ-5D VAS and value set and satisfaction. [file 12955_2019_1237_MOESM6_ESM.docx]

Supplementary Table 6 Detailed description of patient distribution regarding the difference between EQ-5D VAS and value set and satisfaction

| **Difference VAS/EHS-based value set** [**n (%)]** |  |  |  | **Satisfaction (n=135)** | |  |  | |
| --- | --- | --- | --- | --- | --- | --- | --- | --- |
|  |  | **Whole sample (n=137)** |  | **yes (n=53)** | **no (n=82)** |  | **statistics** |  |
| preoperative >0 and postoperative <0 |  | 38 (27.74) |  | 21 (25.61) | 15 (28.30) |  | *X²*=4.60, *df*=3, *p*=0.2033 |  |
| preoperative >0 and postoperative >0 |  | 62 (45.26) |  | 43 (52.44) | 19 (35.85) |  |  |  |
| preoperative <0 and postoperative <0 |  | 23 (16.79) |  | 12 (14.63) | 11 (20.75) |  |  |  |
| preoperative <0 and postoperative >0 |  | 14 (10.22) |  | 6 (7.32) | 8 (15.09) |  |  |  |
|  |  | **preopoperative** |  | **postoperative** |  |  |  |  |
| <0 |  | 37 (27.01) |  | 52 (37.96) |  |  |  |  |
| >0 |  | 100 (72.99) |  | 85 (62.04) |  |  |  |  |
