# Supplementary material for: Patient characteristics and valuation changes impact quality of life and satisfaction in total knee arthroplasty – results from a German prospective cohort study
Source: Health Qual Life Outcomes. 2019 Dec 9;17:180. doi: 10.1186/s12955-019-1237-3 (PMC6902559; doi:10.1186/s12955-019-1237-3)
Supplement: Supplementary file 7 — Additional file 7: Table S7. Comparison of satisfied and not satisfied patients. [file 12955_2019_1237_MOESM7_ESM.docx]

**Supplementary Table 7** Comparison of satisfied and not satisfied patients

|  |  | **satisfied**  **(n=121 (89.63%))** | **not satisfied (n=14 (10.37%))** | **p-value** |
| --- | --- | --- | --- | --- |
| **Age** |  | 70.13 (8.89) | 70.71 (8.27) | 0.83 |
| **Gender male** |  | 49 (40.50%) | 4 (28.57%) | 0.56 |
| **BMI (Mean)** |  | 29.03 (5.89) | 28.65 (5.24) | 0.92 |
| **BMI ≥30** |  | 49 (40.50%) | 5 (35.71%) | 0.78 |
| **Metabolic syndrome (yes)** |  | 6 (4.96%) | 1 (7.14%) | 0.54 |
| **Marital status** | Married | 73 (60.33%) | 9 (64.29%) | 0.92 |
|  | Single | 12 (9.92%) | 1 (7.14%) |  |
|  | Divorced | 5 (4.13%) | 1 (7.14%) |  |
|  | Living Apart | 1 (0.83%) | 0 |  |
|  | Widowed | 30 (24.79%) | 3 (21.43%) |  |
| **Housing situation** | Alone | 38 (31.40%) | 5 (35.71%) | 0.11 |
|  | With partner | 46 (38.02%) | 8 (57.14%) |  |
|  | With family | 31 (25.62%) | 0 |  |
|  | Other | 1 (0.83%) | 0 |  |
|  | . | 5 (4.13%) | 1 (7.14%) |  |
| **Health insurance** | compulsory | 59 (48.76%) | 8 (57.14%) | 0.59 |
|  | private | 62 (51.24%) | 6 (42.86%) |  |
| **Major diagnosis** | right | 66 (54.55%) | 8 (57.14%) | 1.00 |
|  | left | 54 (44.63%) | 6 (42.86%) |  |
|  | bilateral | 1 (0.83%) | 0 |  |
| **Operations at joint before TKR** | 0 | 72 (59.50%) | 8 (57.14%) | 0.94 |
|  | 1 | 34 (28.10%) | 5 (35.71%) |  |
|  | 2 | 11 (9.09%) | 1 (7.14%) |  |
|  | ≥3 | 4 (3.21%) | 0 |  |
| **Cement (cement or hybrid)** | cement | 59 (48.76%) | 7 (50.00%) | 1.00 |
| **Already TKR** |  | 14 (11.57%) | 0 | 0.36 |
| **Already THR** |  | 14 (11.57%) | 0 | 0.36 |
| **Discharge** | home | 24 (19.83%) | 2 (14.29%) | 1.00 |
|  | inpatient rehabilitation | 97 (80.172%) | 12 (85.71%) |  |
| **Charlson Comorbidity Index** | 0 | 79 (65.29%) | 8 (57.14%) | 0.29 |
|  | 1 | 32 (26.45%) | 4 (28.57%) |  |
|  | 2 | 3 (2.48%) | 2 (14.29%) |  |
|  | ≥3 | 7 (5.79%) | 0 |  |
| **ASA Physical Score Classification** | 1 | 29 (23.97%) | 4 (28.57%) | 0.92 |
|  | 2 | 76 (62.81%) | 8 (57.14%) |  |
|  | 3 | 16 (13.22%) | 2 (14.29%) |  |
| **Infiltration anaesthesia** |  | 40 (33.06%) | 4 (28.57%) | 1.00 |
| **FNB/ASNB/SSNB** |  | 57 (47.11%) | 6 (42.86%) | 0.79 |
| **PDA** |  | 8 (6.61%) | 0 | 1.00 |
| **Preoperative hemoglobin** |  | 13.96 (1.14) | 13.69 (1.28) | 0.51 |
| **Number of operations and other procedures** |  | 2.00 (0.87) | 2.00 (1.66) | 0.17 |
| **Knee Society Score** |  | 52.14 (16.53) | 54.64 (15.02) | 0.82 |
| **Knee Society Score function** |  | 66.24 (20.74) | 69.29 (22.94) | 0.50 |
| **EQ-5D value set (preoperative)** |  | 0.52 (0.15) | 0.54 (0.17) | 0.82 |
| **EQ-5D VAS (preoperative)** |  | 61.71 (19.86) | 63.79 (15.72) | 0.83 |
| **WOMAC pain (preoperative)** |  | 55.02 (18.41) | 55.29 (15.72) | 0.88 |
| **WOMAC stiffness (preoperative)** |  | 46.61 (23.32) | 50.00 (24.57) | 0.75 |
| **WOMAC function (preoperative)** |  | 53.39 (20.37) | 58.40 (14.82) | 0.37 |
| **WOMAC sum (preoperative)** |  | 53.16 (19.3) | 57.05 (14.23) | 0.46 |
