# Supplementary material for: Patient characteristics and valuation changes impact quality of life and satisfaction in total knee arthroplasty – results from a German prospective cohort study
Source: Health Qual Life Outcomes. 2019 Dec 9;17:180. doi: 10.1186/s12955-019-1237-3 (PMC6902559; doi:10.1186/s12955-019-1237-3)
Supplement: Supplementary file 8 — Additional file 8: Table S8. Linear regression model predicting postoperative WOMAC Sum. [file 12955_2019_1237_MOESM8_ESM.docx]

Supplementary Table 8 Linear regression model predicting postoperative WOMAC Sum

|  |  | **Postoperative WOMAC Sum** |
| --- | --- | --- |
|  |  | Estimate [95% CI] |
| Intercept |  | 42.42** [13.59 - 71.24] |
| Preoperative difference VAS/EHS-based value set |  | 0.21** [0.06 – 0.37] |
| Preoperative WOMAC sum |  | 0.16* [0.02 – 0.29] |
| Age |  | 0.39* [0.04 – 0.73] |
| Gender, male |  | 0.61 [-5.75 – 6.97] |
| Marital status | Married | Ref. |
|  | Single | 6.03 [-6.34 – 18.40] |
|  | Divorced | -10.22 [-26.08 – 5.65] |
|  | Living Apart | -1.89 [-29.07 – 25.29] |
|  | Widowed | -3.29 [-38.22 – 31.64] |
| Housing situation | Alone | Ref. |
|  | With partner | -0.86 [-13.49 – 11.78] |
|  | With family | 8.13 [-5.34 – 21.61] |
|  | Other | -3.29 [-38.22 – 31.64] |
| Charlson Comorbidity Index | 0 | Ref. |
|  | 1 | -0-38 [-6.91 – 6.15] |
|  | 2 | -13-16 [-27.27 – 0.95] |
|  | ≥3 | -2-10 [-14.31 -10.11] |
| ASA Physical Score Classification | 1 | Ref. |
|  | 2 | -3.30 [-10.16 – 3.56] |
|  | 3 | -1.40 [-13.23 – 10.43] |
| Number of side diagnosis |  | 0.49 [-0.75 – 1.73] |

*p<0.05 **p<0.01 ***p<0.001
