# Supplementary material for: Patient characteristics and valuation changes impact quality of life and satisfaction in total knee arthroplasty – results from a German prospective cohort study
Source: Health Qual Life Outcomes. 2019 Dec 9;17:180. doi: 10.1186/s12955-019-1237-3 (PMC6902559; doi:10.1186/s12955-019-1237-3)

Supplementary Figure 1 Scatterplots of WOMAC sum/EQ-5D VAS change/postoperative scores by preoperative scores, grouped by patient satisfaction.

The horizontal line depicts patient acceptable symptom state (PASS). Highest levels of satisfaction (Likert scale 9 and 10) were defined as satisfied.


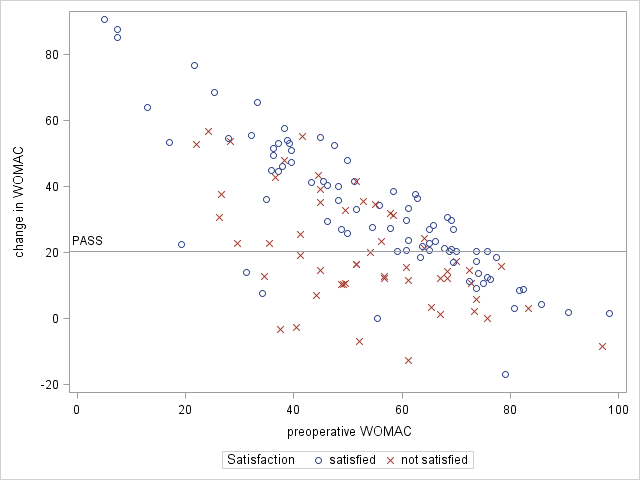

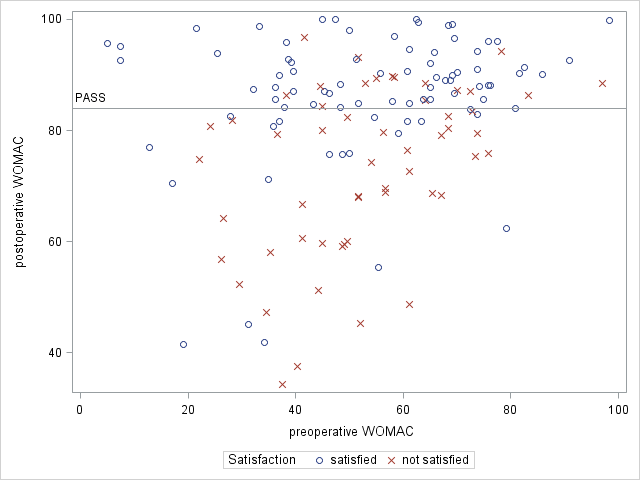

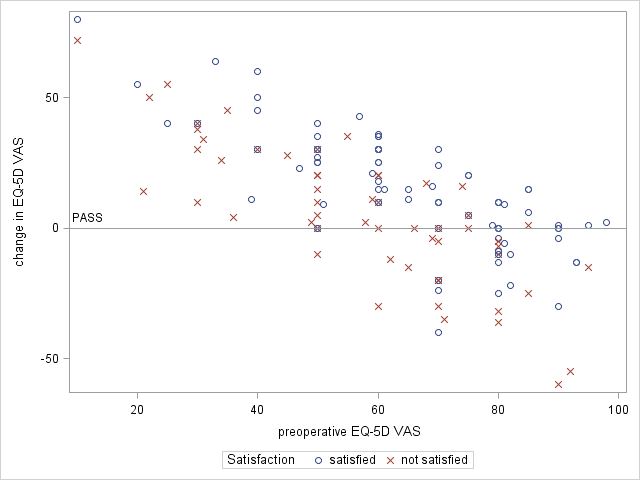

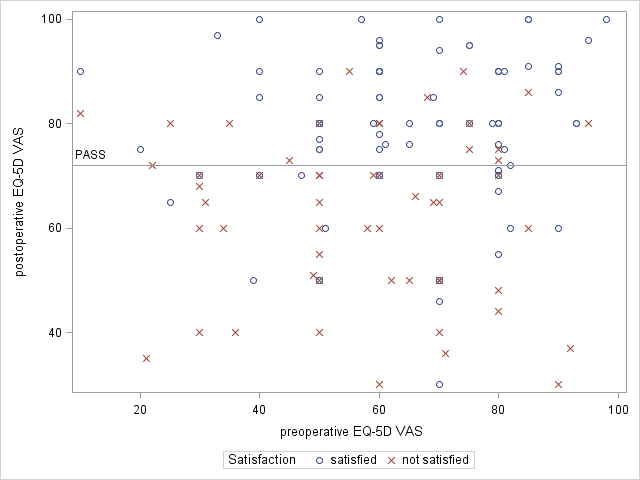

Supplement: Supplementary file 9 — Additional file 9: Figure S1. Scatterplots of WOMAC sum/EQ-5D VAS change/postoperative scores by preoperative scores, grouped by patient satisfaction. [file 12955_2019_1237_MOESM9_ESM.docx]
